# Familial Hypocalciuric Hypercalcemia in Pregnancy: Diagnostic Pitfalls

**DOI:** 10.1002/jbm4.10362

**Published:** 2020-04-27

**Authors:** Alicia R Jones, Matthew JL Hare, Justin Brown, Jun Yang, Caroline Meyer, Frances Milat, Carolyn A Allan

**Affiliations:** ^1^ Monash Centre for Health Research and Implementation Monash University Melbourne Australia; ^2^ Department of Endocrinology Monash Health Melbourne Australia; ^3^ Wellbeing and Chronic Preventable Diseases Division Menzies School of Health Research Darwin Australia; ^4^ Department of Paediatrics Monash University Melbourne Australia; ^5^ Department of Paediatric Endocrinology and Diabetes Monash Health Melbourne Australia; ^6^ Hudson Institute of Medical Research, Clayton Melbourne Australia; ^7^ Department of Medicine Monash University Melbourne Australia; ^8^ Department of Endocrinology St Vincent's Hospital Melbourne Australia; ^9^ Department of Obstetrics and Gynaecology Monash University Melbourne Australia

**Keywords:** DISORDERS OF CALCIUM/PHOSPHATE METABOLISM, GENETICS, PARATHYROID HORMONE

## Abstract

Familial hypocalciuric hypercalcemia (FHH) is a group of autosomal dominant disorders caused by dysfunction of the calcium sensing receptor (CaSR) and its downstream signaling proteins, leading to generally asymptomatic hypercalcemia. During pregnancy, distinguishing FHH from primary hyperparathyroidism (PHPT) is important, as the latter is associated with adverse outcomes and can be treated surgically during pregnancy, whereas the former is benign. This case report highlights the difficulties in diagnosing FHH during pregnancy. A 32‐year‐old woman was found to have asymptomatic hypercalcemia at 14‐weeks’ gestation. Investigations showed a corrected calcium (cCa) of 2.61 mmol/L (2.10 to 2.60), ionized Ca (iCa) of 1.40 mmol/L (1.15 to 1.28), 25OHD of 33 nmol/L (75 to 250), and PTH of 9.5 pmol/L (1.5 to 7.0). The patient was treated with 2000 IU cholecalciferol daily with normalization of 25OHD. The urine calcium / creatinine clearance ratio (CCCR) was 0.0071, and neck US did not visualize a parathyroid adenoma. Upon a retrospective review of the patient's biochemistry from 2 years prior, hypercalcemia was found that was not investigated. The patient was monitored with serial iCa levels and obstetric US. She delivered a healthy boy at 38‐weeks’ gestation. Postnatal iCa was 1.48 mmol/L and remained elevated. Her son had elevated iCa at birth of 1.46 mmol/L (1.15 to 1.33), which rose to 1.81 mmol/L by 2 weeks. He was otherwise well. Given the familial hypercalcemia, a likely diagnosis of FHH was made. Genetic testing of the son revealed a missense mutation, NM_000388.3(CASR):c.2446A > G, in exon 7 of the CaSR, consistent with FHH type 1. To our knowledge, there are only three existing reports of FHH in pregnancy. When differentiating between FHH and PHPT in pregnancy, interpretation of biochemistry requires an understanding of changes in Ca physiology, and urine CCCR may be unreliable. If the decision is made to observe, clinical symptoms, calcium levels, and fetal US should be monitored, with biochemistry and urine CCCR performed postpartum, once lactation is completed © 2020 The Authors. *JBMR Plus* published by Wiley Periodicals, Inc. on behalf of American Society for Bone and Mineral Research.

## Case Summary

A 32‐year‐old woman was referred to our endocrine pregnancy clinic for hypercalcemia, incidentally detected on her first trimester screening, at 14‐weeks’ gestation. She had no past medical history and her medications included a multivitamin and cholecalciferol 1000 IU daily. She was gravida 2, parity 1, with a healthy 2‐year‐old daughter. She had mild nausea and lethargy, but no polyuria, bony pain, constipation, or nephrolithiasis. Her physical examination was unremarkable, with no palpable neck masses. There was no known family history of calcium disorders.

Initial investigations showed corrected calcium (cCa) of 2.61 mmol/L (2.10 to 2.60), 25OHD of 33 nmol/L (75 to 250), and elevated PTH of 9.5 pmol/L (1.5 to7.0). Repeat biochemistry early in the second trimester showed ionized calcium (iCa) of 1.40 mmol/L (1.15 to1.28), phosphate of 0.86 mmol/L (0.80 to1.50), and magnesium of 0.73 mmol/L (0.70 to1.10); all other electrolytes were normal (Table 1). Her 1‐25‐dihydroxyvitamin D level was not assessed. The 24‐hour urine calcium / creatinine clearance ratio (CCCR) was 0.0071. Neck US did not visualize a parathyroid adenoma. A retrospective review of biochemistry from the first trimester of her first pregnancy 2 years prior demonstrated an elevated cCa of 2.87 mmol/L that had not been investigated.

**Table 1:** Maternal Biochemistry Over Time

	Nov 2015	June 2017	Sept 2017	Nov 2017	Jan 2018 (birth)	Feb 2018	Sept 2018	Dec 2018
Trimester	Third	First	Second	Third	Postpartum			
iCa (1.15 to 1.33) mmol/L			1.40	1.46	1.48	1.42		
cCa (2.10 to 2.60) mmol/L	2.87	2.61	2.72	2.68	2.77	2.93	2.82	2.74
PTH (1.5 to 7.0) pmol/L		9.5	7.6	4.9		9.4	10.4	
PO_4_ (0.80 to 1.5) mmol/L	0.70		0.86	0.85	0.89	0.79	0.94	1.03
Cr (45 to 90) μmol/L		72	63	45		59	67	69
ALP (30 to 110) U/L		52	56		152	79		65
25(OH)VitD (75 to 250) nmol/L	74	33	60	54		66	37	
24‐hour urine CE (2.0 to 5.0)		4.8				1.52		
24‐hour urine CCR (<0.40)		0.30				0.11		
24‐hour urine CCCR		0.0071				0.0024		

iCa = ionized calcium; cCa = corrected calcium; PTH = parathyroid hormone; PO_4_ = phosphate; Cr = creatinine; ALP = alkaline phosphatase; 25(OH)Vit D = 25‐hydroxyvitamin D; CE = calcium excretion; CCR = calcium to creatinine ratio; CCCR = calcium to creatinine clearance ratio.

The patient was treated with 2000 IU cholecalciferol oral daily for the remainder of the pregnancy, and a repeat 25OHD level at 17‐weeks’ gestation was 60 nmol/L, with a PTH of 7.6 pmol/L. The patient was monitored with serial iCa levels during pregnancy and these remained stable (Table 1). Obstetric US at 37‐weeks’ gestation showed growth restriction with an estimated fetal weight on the 15^th^ percentile. She had an induced labor at 38‐ + 3‐weeks’ gestation, and delivered a healthy boy. Postnatal iCa was 1.48 mmol/L, and calcium has remained elevated since then.

Her son had a birthweight of 2600 g and Apgar scores of 8 at 1 min and 9 at 5 min. His iCa at birth was elevated at 1.46 mmol/L (1.15 to 1.33); however, he was otherwise well and examined normally, so this was not treated. Repeat biochemistry of the son at 2 weeks of age found an elevated iCa of 1.81 mmol/L and cCa of 3.29 mmol/L (2.20 to 2.80), with a low PTH of 0.6 pmol/L (1.5 to 7.0). He was feeding well with normal growth. Given the magnitude of the hypercalcemia, he was admitted for i.v. hydration and changed to low calcium formula feeds. His calcium declined over subsequent days, but remained elevated; his PTH increased to 8.2 pmol/L (Table 2).

**Table 2:** Son's Biochemistry Over Time

	Jan 2018 (birth)	Feb 2018	Feb 2018	March 2018	April 2018	Sep 2018	Dec 2018
iCa mmol/L (1.15 to 1.33)	1.46	1.81	1.45	1.66	1.73	1.58	1.58
cCa mmol/L (<7days: 2.85 to 2.80) (7days to <6 months: 2.20 to 2.80) 6 months to <2 years: 2.20 to 2.70)		3.29	2.95	3.02	3.22	3.10	3.06
PTH pmol/L (1.5 to 7.0)		0.6	8.2	4.7	1.0	3.1	3.9
PO_4_ mmol/L (<7days: 1.25 to 2.85) (7 days to 28 days 1.50 to 2.75) (1 month to <6 months: 1.45 to 2.50) (6 months to <1year: 1.30 to 2.30)	1.87	1.50	2.02	1.77	1.47	1.63	1.45
Cr μmol/L (<7days: 22 to 93) (7 days to 27 days: 17 to 50) (28 days to <2 years: 11 to 36)		15					
ALP U/L (<28 days: 80 to 550) (28 days to <6 months: 120 to 650) (6 months to <2 years: 120 to 450)			362	519	564	263	306
25(OH)VitD nmol/L (75 to 250)		46					89
Spot urine CCR mol/mol (<1year: <1.5)		0.10	1.20	2.00		0.50	0.20

iCa = ionized calcium; cCa = corrected calcium; PTH = parathyroid hormone; PO_4_ = phosphate; Cr = creatinine; ALP = alkaline phosphatase; 25(OH)Vit D = 25‐hydroxyvitamin D; CCR = calcium / creatinine ratio.

Given that both mother and son had asymptomatic hypercalcemia, a likely diagnosis of familial hypocalciuric hypercalcemia (FHH) was made. Subsequent investigations showed mild hypercalcemia (cCa 2.49 mmol/L) in the patient's 2‐year‐old daughter; however, her partner and parents had normal calcium levels, and no other family members were tested. Genetic testing of the son revealed a missense mutation, NM_000388.3(CASR):c.2446A > G:p.(lle816Val), in exon 7 of the calcium sensing receptor (CaSR), consistent with FHH type 1.

## Discussion

FHH is a group of autosomal dominant disorders caused by dysfunction of the CaSR and its downstream signaling proteins, characterized by chronic, nonprogressive hypercalcemia, which is generally asymptomatic.^1^ Differentiation from primary hyperparathyroidism (PHPT) is important, but can be challenging, especially during pregnancy. To our knowledge, there are only three other case reports of FHH in pregnancy, with possible pre‐existing diagnosis in one case.

FHH type 1 is caused by an inactivating mutation in the gene encoding the CaSR and accounts for the majority of cases. FHH type 2 occurs as a result of an inactivating mutation in the G protein subunit alpha 11 (GNA11) gene encoding subunit α_11,_ which mediates intracellular signaling from the CaSR. FHH type 3 is caused by mutations of the adaptor‐related protein complex 2 subunit sigma 1 (AP2S1) gene, which encodes the adaptor‐related protein 2 complex that facilitates endocytosis of the CaSR.^1^ The mutation in our patient's son, NM_000388.3(CASR):c.2446A > G:p.(lle816Val), causes a change from the amino acid isoleucine to valine at position 816 of the protein, inactivating the CaSR. This has previously been described in a family with hypocalciuric hypercalcemia.^2^

The CaSR is expressed in the parathyroid glands and kidneys, as well as many other organs (including bone, placenta, brain, breast)^3^ and regulates PTH secretion and renal excretion of calcium. Patients with FHH have lifelong mild to moderately elevated calcium levels, normal or mildly elevated PTH levels, and reduced urine calcium excretion.^4^ It is thought to be a benign condition, with no definite association with adverse outcomes.^5^, ^6^, ^7^, ^8^ Importantly, there is no specific treatment for FHH. Parathyroidectomy will not restore calcium levels to normal. This makes differentiation between FHH and PHPT important, as the latter is associated with osteoporosis, nephrolithiasis, and pancreatitis, and easily treatable by parathyroidectomy, preferably during the second trimester.^9^, ^10^ Subsequent confirmation of FHH type 1 in this family supported the decision to closely monitor the patient and not perform surgery during the pregnancy. Had the genetics been confirmed earlier, the son arguably may not have required rehospitalization in the neonatal period.

In pregnancy, PHPT has been associated with maternal pre‐eclampsia, pancreatitis, miscarriage, and hypercalcemic crisis, as well as fetal intrauterine growth retardation, parathyroid hypoplasia, and subsequent neonatal hypocalcemia.^11^ The risk of these events may be related to the severity of hypercalcemia.^12^, ^13^ Three cases of FHH in pregnancy have been previously described.^14^, ^15^, ^16^, ^17^ Two cases had presented with hypercalcemia several years prior to pregnancy, with possible genetic confirmation prior to pregnancy in one of the cases.^14^, ^15^ No adverse maternal outcomes were reported, although one patient underwent unnecessary neck exploration and three‐gland parathyroidectomy, as FHH was diagnosed postpartum.^16^, ^17^ There were also no adverse fetal outcomes; however, the offspring in these limited cases were genetically concordant with the mothers.^14^, ^15^, ^16^, ^17^ Neonatal biochemistry in FHH will depend on the genotype. Unaffected neonates are at risk of hypocalcemia initially after birth because of parathyroid gland suppression in utero. Heterozygous neonates born to an affected mother will have hypercalcemia. Heterozygous neonates born to an unaffected mother may have severe hypercalcemia caused by secondary hyperparathyroidism in utero. An homozygous or compound heterozygous neonate is at risk of neonatal severe hyperparathyroidism, requiring urgent parathyroidectomy.^18^, ^19^

Distinguishing between FHH and PHPT can be difficult, as 20% of those with FHH have elevated PTH levels, and 10% to 20% of those with PHPT have normal PTH levels.^1^ During pregnancy, interpretation is further complicated by changes in calcium physiology. Dilutional hypoalbuminemia causes a reduction in total calcium levels, although iCa and cCa remain relatively stable. Placental lactogen and PTH‐related peptide (PTHrP), released from the placenta and breast, act to stimulate 1‐alpha hydroxylase activity, which increases levels of 1,25(OH)_2_D_3_. The maternal kidneys are the primary source of 1‐alpha hydroxylase, with a relatively minor contribution from placental 1‐alpha hydroxylase.^20^ Negative feedback on the parathyroid glands from PTHrP reduces PTH levels. Both 1,25(OH)_2_D_3_ and PTHrP act on the skeleton to increase bone turnover, and 1,25(OH)_2_D_3_ acts on the intestines to increase the absorption of calcium, which doubles from around the 12th week of gestation.^21^ This increase in intestinal absorption causes an increase in 24‐hour urine calcium excretion, termed absorptive hypercalciuria, although fasting levels may be normal or even low, highlighting the role of intestinal absorption.^21^ One study found that 24‐hour urine calcium excretion was 250% to 300% higher in pregnancy when compared with postpartum excretion.^22^

The CCCR, based on paired serum and 24‐hour urine levels, is considered the best test to differentiate FHH from PHPT. Findings presented at the Fourth International Workshop on PHPT proposed that a CCCR <0.01 is suggestive of FHH, CCCR >0.02 makes FHH unlikely, and a CCCR of 0.01 to 0.02 is uncertain, with genetic testing indicated in such cases.^23^ There is considerable overlap in the CCCR between FHH and PHPT. A Danish study of 54 patients with genetically confirmed FHH and 97 patients with histologically proven PHPT, found sensitivity of 80% and specificity of 88% for diagnosing FHH, using a urine CCCR cut‐off of <0.0112. Using a cut‐off of <0.027 improved the sensitivity to 100%, but reduced specificity to 65%.^24^

Unfortunately, the absorptive hypercalciuria of pregnancy makes interpretation of urine CCCR difficult. Of the three existing case reports of FHH in pregnancy, only two had urine CCCR reported, with values of 0.0199 and 0.019, both within the range considered uncertain in nonpregnant individuals (Table 3).^15^, ^16^ Other tests used to distinguish FHH and PHPT, such as fasting urine calcium excretion (CE, urine Ca / urine creatinine × serum creatinine) and urine calcium / creatinine excretion ratio (24‐hour urine calcium excretion / 24‐hour urine creatinine excretion), have lower specificity in nonpregnant populations, and again have not been studied in pregnancy.^24^ Comorbid vitamin D deficiency, as in this case, will increase PTH levels, and reduce urine CE, making interpretation of biochemistry more difficult. A new risk equation for FHH, Pro‐FHH, using calcium, PTH, urine CCCR, and bone remodeling markers, was recently published; however, it has not been validated in pregnancy.^25^ Nuclear medicine imaging is contraindicated during pregnancy, and neck US is operator‐dependent, with no known sensitivity and specificity for use during pregnancy.^26^ Genetic testing is expensive, not widely available, and the results may not be available in a suitable timeframe to be useful in pregnancy, but it is recommended if available. Past biochemistry and family history may be helpful when available, as in our case, but can neither exclude nor confirm the diagnosis. The mild hypercalcemia observed in the mother's first child could have increased suspicion of FHH in this family, had this been performed during pregnancy. The limitations of various tests in differentiating between FHH and PHPT in pregnancy are listed in Table 4.

**Table 3:** Biochemistry From Existing Case Reports of FHH During Pregnancy

Case	Peak cCa mmol/L	PTH pmol/L	Urine CCCR	Urine CE mmol/L
Murthy et al., 2009^14^	3.35	7.3	n/a	2.4
Ghaznavi et al., 2016^15^	2.59	4.9	0.0199	n/a
Maltese et al., 2017^16^ and Walker et al., 2014^17^; both report on the same patient.	3.01	3.7	0.019	8.6

FHH = Familial hypocalciuric hypercalcemia; cCa = corrected calcium; PTH = parathyroid hormone; CCCR = calcium / creatinine clearance ratio; CE = calcium excretion; n/a = not available.

**Table 4:** Investigations to Differentiate FHH From PHPT and Their Limitations in Pregnancy

Investigation	Limitations in pregnancy
24‐hour urine CCCR	False‐negative in pregnancy caused by absorptive hypercalciuria No pregnancy reference ranges
Fasting urine CE	Sensitivity and specificity lower than urine CCCR outside of pregnancy No pregnancy reference ranges
Neck ultrasound	Limited accuracy for parathyroid adenoma Operator‐dependent
Family history	May have no known family history Familial forms of both FHH and PHPT exist
Genetic testing	Delay in results

FHH = Familial hypocalciuric hypercalcemia; PHPT = primary hyperparathyroidism; CCCR = calcium / creatinine clearance ratio; CE = calcium excretion.

## Conclusion

In practice, diagnosing FHH in pregnancy requires a high level of suspicion. In women with newly diagnosed hypercalcemia in pregnancy, we recommend a multidisciplinary approach involving the endocrinology, obstetrics, and pediatrics departments. When differentiating between FHH and PHPT in pregnancy, the interpretation of biochemistry requires an understanding of calcium physiology in pregnancy, and physicians should be aware that the urine CCCR may be unreliable. If the decision is made to observe, clinical symptoms, calcium levels, and fetal US should be monitored closely, with biochemistry and urine CCCR performed postpartum and once lactation is completed. Confirmation of FHH with genetic testing, as in this case, provided additional reassurance in the postpartum period, and should be performed if available during the pregnancy.

## Disclosures

No authors have any disclosures to declare.
